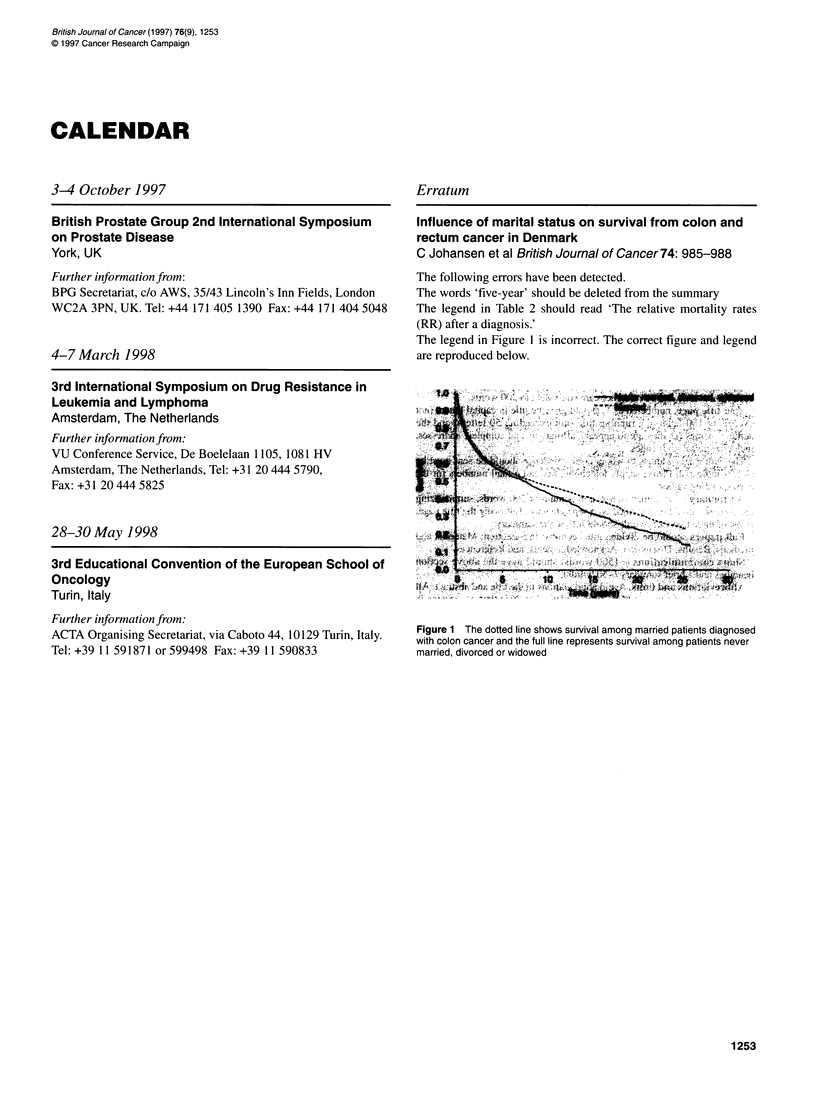# Calendar

**Published:** 1997

**Authors:** 


British Journal of Cancer (1997) 76(9), 1253
© 1997 Cancer Research Campaign

### CALENDAR

### *3–4 October 1997*

**British Prostate Group 2nd International Symposium on Prostate Disease**
York, UK

*Further information from*:
BPG Secretariat, c/o AWS, 35/43 Lincoln's Inn Fields, London WC2A 3PN, UK. Tel: +44 171 405 1390 Fax: +44 171 404 5048

### *4–7 March 1998*

**3rd International Symposium on Drug Resistance in Leukemia and Lymphoma**
Amsterdam, The Netherlands

*Further information from:*
VU Conference Service, De Boelelaan 1105, 1081 HV Amsterdam, The Netherlands, Tel: +31 20 444 5790, Fax: +31 20 444 5825

### *28–30 May 1998*

**3rd Educational Convention of the European School of Oncology**
Turin, Italy

*Further information from:*
ACTA Organising Secretariat, via Caboto 44, 10129 Turin, Italy. Tel: +39 11 591871 or 599498 Fax: +39 11 590833